# A Small In Vitro Fermentation Model for Screening the Gut Microbiota Effects of Different Fiber Preparations

**DOI:** 10.3390/ijms20081925

**Published:** 2019-04-18

**Authors:** Irina Tsitko, Fanny Wiik-Miettinen, Outi Mattila, Natalia Rosa-Sibakov, Tuulikki Seppänen-Laakso, Johanna Maukonen, Emilia Nordlund, Maria Saarela

**Affiliations:** 1 VTT Technical Research Centre of Finland, Limited, Post Office Box 1000, Tietotie 2, 02044 Espoo, Finland; fanny.wiik@helsinki.fi (F.W.-M.); outi.mattila@vtt.fi (O.M.); natalia.rosa-sibakov@vtt.fi (N.R.-S.); Tuulikki.Seppanen-Laakso@vtt.fi (T.S.-L.); pia-johanna.maukonen@dupont.com (J.M.); emilia.nordlund@vtt.fi (E.N.); mariahsaarela@gmail.com (M.S.); 2 Current address: DuPont Nutrition & Health, 02460 Kantvik, Finland

**Keywords:** inulin, resistant starch, rye bran, fiber preparation, linseed, in vitro colon model, fecal fermentation

## Abstract

The development of prebiotic fibers requires fast high-throughput screening of their effects on the gut microbiota. We demonstrated the applicability of a mictotiter plate in the in vitro fermentation models for the screening of potentially-prebiotic dietary fibers. The effects of seven rye bran-, oat- and linseed-derived fiber preparations on the human fecal microbiota composition and short-chain fatty acid production were studied. The model was also used to study whether fibers can alleviate the harmful effects of amoxicillin-clavulanate on the microbiota. The antibiotic induced a shift in the bacterial community in the absence of fibers by decreasing the relative amounts of Bifidobacteriaceae, Bacteroidaceae, Prevotellaceae, Lachnospiraceae and Ruminococcaceae, and increasing proteobacterial Sutterilaceae levels from 1% to 11% of the total microbiota. The fermentation of rye bran, enzymatically treated rye bran, its insoluble fraction, soluble oat fiber and a mixture of rye fiber:soluble oat fiber:linseed resulted in a significant increase in butyrate production and a bifidogenic effect in the absence of the antibiotic. These fibers were also able to counteract the negative effects of the antibiotic and prevent the decrease in the relative amount of bifidobacteria. Insoluble and soluble rye bran fractions and soluble oat fiber were the best for controlling the level of proteobacteria at the level below 2%.

## 1. Introduction

The gut microbiota has been shown to have a remarkable impact on the overall health status of the host organism. Main external factors that can affect the composition of the gut microbiota in generally healthy people include antibiotic therapy and major dietary changes. Antibiotic treatments are known to have negative short-term as well as long-term side effects on commensal gut microbiota. Different kinds of disturbances of the gut microbiota upon antibiotic challenges have been reported, from the reduction in a total number of gut bacteria to the changes in relative amounts of particular bacterial groups, metabolic dysfunction, and antibiotic-associated diarrhea as a common side effect [1,2,3,4,5].

Intervention with dietary fibers is one of the attractive options to modulate the gut microbiota [6,7]. Physiological effects of dietary fiber are dependent on their physicochemical properties, which are mainly influenced by the particle size, cell wall architecture, solubility, degree of polymerization and substitution, distribution of side chains and degree of cross-linking of the polymers [7,8]. Thus, well designed functional fibers could selectively target beneficial bacterial groups in the gut microbial population.

Among the gut microbial population, the main saccharolytic genera, i.e., the bacterial groups metabolizing dietary fibers in the human gastrointestinal tract are *Bacteroides, Bifidobacterium, Clostridium, Eubacterium, Lactobacillus*, and *Ruminococcus* [9]. The saccharolytic genera are able to produce short chain fatty acids (SCFA) which have both local and systemic beneficial biological effects on the host. Overall, SCFAs decrease the pH in the colon, which hampers pathogenic bacterial colonization and growth as well as activates the immune response of the host [10,11]. Propionate has been shown to inhibit cholesterol synthesis and butyrate, additionally to serving as a fuel for colon epithelial cells, and has been proposed to play an important role as a protective agent against chronic intestinal inflammation [12,13,14]. In addition to the beneficial metabolites from dietary fibers, prebiotic fibers could potentially reduce the negative effect of antibiotics on the gut microbiota by supporting the growth of the beneficial bacterial groups, and consequently, alleviating the symptoms the patients experience during the antibiotic therapy [15,16]. 

The development of new fibers requires frequent testing in a feasible way. Human, as well as animal model experiments, are laborious and limited by the cost and ethical issues. Moreover, in vivo animal modes, used to study human microbiota, still cannot reflect the whole complicity of the human digestive system environment. In vitro gut microbiota models have proved to be useful tools to study the effects of food components, probiotics, and pharmaceutical molecules on gut microbiota composition. The advantages and limitations of in vivo and in vitro models as well as the developments to improve the modeling of host-microbe interactions have been extensively reviewed [17,18,19,20,21]. Generally, compared to human or animal experiments, in vitro models are cheaper, can be performed under standardized conditions, easier to control and repeat. Although in vivo models cannot provide complete host factors, they are valuable tools for studying and uncovering microbiota response to different compounds. 

Since the digestion of complex carbohydrates, which are not processed in the small intestine, takes place in the colon [22], the colon model is the most suitable tool for studying the potential prebiotic properties of the dietary fibers. Various types of models have been developed to study the effect of different compounds on human gut microbiota and its metabolic functions. There are two main types of models, continuous and static cultures, varying in complicity, ranging from a simple batch to complex multistage continuous culture systems [17,18,19,20]. Multistage continuous models are designed to mimic a specific part of the gastrointestinal tract, digestion dynamic, pH changes, the transition time of chyme and secretion of digestive enzymes, bile salt and nutrients [23,24,25,26,27]. The simplest version of in vitro models is a static batch fermentation with fecal slurry or feces-derived microbial populations [19,28]. Static fermentation models do not reflect adequately the real colon environment, where the produced SCFAs, vitamins and small metabolites are absorbed by the epithelial cells. Therefore, they are limited to short-term experiments. However, since most of the multistage continuous models use large volumes, require sophisticated on-line monitoring, are laborious, time-consuming, and have a relatively low throughput, the static models are considered as an attractive alternative for initial screening stages.

The current trend in in vitro model development is to establish small volume flexible systems. A recent example is a semi single-stage colon model CoMiniGut with pH and temperature control [29]. Yet, in this model only a small number of parallels can be run at the same time. Microtiter plate format fermentation systems could facilitate the screening of a large number of compounds/dietary fibers, which is essential especially at the initial stage of the development of new dietary fibers. Very recently described the colon model based on 24-well microtiter plate showed a high level of reproducibility [30]. 

The aim of our study was to establish a small format in vitro batch colon model as a screening tool for the effects of dietary fibers and their combinations on gut microbiota composition and SCFA production. We have investigated whether the system can be useful in the screening of the experimental fibers. Further, the model was used to test the hypothesis that the application of specific dietary fiber preparations will help to support the recovery and growth of commensal gut bacteria during antibiotic treatment.

## 2. Results

### 2.1. Fiber Characteristics

In total, eleven dietary fiber preparations were used in the study, including seven experimental samples as presented in Table 1. While most of the dietary fibers in rye bran-derived samples and linseed were insoluble, oat β-glucan was mainly soluble. Insoluble fraction of enzymatically treated rye bran (Insol EnzRB) and SolBG sample (soluble oat fiber preparation) contained the highest amount of dietary fiber, and the Insol EnzRB had the highest content of insoluble fiber whereas solBG had the highest content of soluble fiber. Soluble enzymatically prepared rye bran fraction was characterized by high content of easily available sugars. Linseed preparation had the highest amount of protein.

In addition to the seven experimental dietary fiber preparations, commercial dietary fiber preparations, including microcrystalline cellulose, resistant starch and xylo-oligosaccharide (XOS) and inulin were included in the study (Table 2).

### 2.2. Fecal Fermentations of Commercial Dietary Fibers

Commercial dietary fiber preparations (Table 2), microcrystalline cellulose (MCC), resistant starch (RS) and xylo-oligosaccharide (XOS) and inulin were studied in the developed in vitro model. Both the control without added carbon source and microcrystalline cellulose (MCC) caused a small increase in the numbers of bifidobacteria (estimated with qPCR) compared to the baseline, which could result from the presence of fermentable carbohydrates in simulated ileal efflux medium (SIEM).

**Figure 1:**
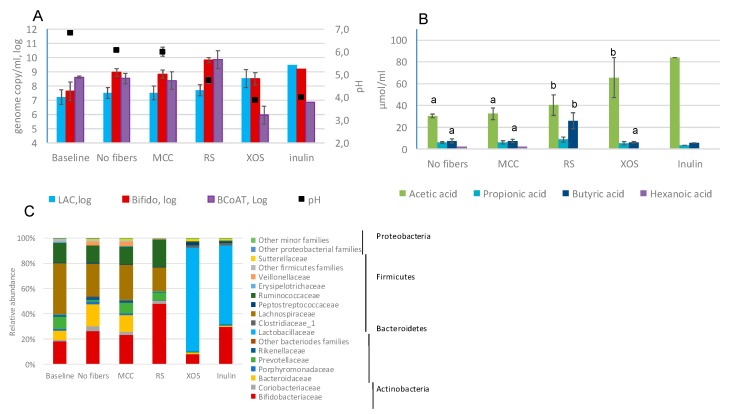
The bacterial population changes and SCFA accumulation after 48 h fecal fermentation of commercial dietary fiber preparations. (**A**) The number of main microbial groups as defined by qPCR; (**B**) the concentration of SCFAs at the end on the fermentation; (**C**) bacterial community profile at family level. LAC—*Lactobacillus* group, Bifido—*Bifidobacterium* spp., BCoAT—butyryl-CoA:acetate CoA-transferase gene; MCC—microcrystalline cellulose, RS—resistant starch, XOS—xylo-oligosaccharide. Values with different letters showed significant differences among the groups (*p* < 0.05). The samples with inulin were not included in analysis of variance (ANOVA) since only duplicates were available for the analysis.

Fecal fermentation of XOS and inulin resulted in an increase in the level of *Lactobacillus* group (Figure 1A). After 48 h fermentation of these fibers, the number of lactic acid bacteria as quantified by qPCR was 1–2 log higher than in the presence of MCC. Resistant starch was bifidogenic; the level of bifidobacteria was 1 log higher than in the model with MCC or with no fibers. Out of tested fibers, only resistant starch promoted the growth of butyrate-producing bacteria (Figure 1A). With all tested four fibers the final pH was maintained above 4.0. 

All four fibers resulted in an increase of total short-chain fatty acids (SCFA) as compared to MCC (51 µmol/mL with MCC vs. 74–93.4 µmol/mL with the dietary fibers). Acetic acid production was the highest with inulin (83.7 µmol/mL), followed by (61.6 µmol/mL), XOS (65.5 µmol/mL) and resistant starch (40.5 µmol/mL). The highest accumulation of butyric acid (26 µmol/mL) and propionic acid (8.8 µmol/mL) was detected with resistant starch (Figure 1B). 

The overall bacterial community composition was analyzed using 16S rRNA gene amplicon sequencing. The sequencing yielded 5,338,637 high quality reads with an average of 108,951 reads per sample. For the analysis, all data were normalized to 57,210 reads per sample. 

The bacterial community in the pooled fecal slurry (baseline) was dominated by Firmicutes (60.1% of total bacteria), Actinobacteria and Bacteroidetes (19.5% each) (Figure 1C). The most dominant families were Lachnospiraceae (40.8% of total bacteria), followed by Bifidobacteriaceae (18%), Ruminococcaceae (15.4%), Prevotellaceae (9.7%) and Bacteroidaceae (7.1%). Proteobacteria constituted the minor part (0.6%) and all other phyla accounted for less than 0.5% of the total bacterial community. The bacterial composition on the genus level is presented in Figure S1. 

The bacterial composition in the control samples (without fibers and with MCC) after 48 h incubation differed from that at the baseline. There was an increase in the relative abundance of Bifidobacteriaceae, Bacteroidaceae and Coriobacteriaceae, while the level of Lachnospiraceae decreased from 40% in the baseline to 25–27.5% at the end of the 48 h fermentation. The bacterial compositions in the samples incubated with MCC were similar to samples incubated without any fibers after 48 h of fermentation.

After 48 h of fermentation of XOS and inulin, the main family detected was Lactobacillaceae (71.3%, 87.2%, and 62%, respectively). Supplementation with these two fibers resulted in a low abundance of Lachnospiraceae and Ruminococcaceae. Inulin led also to an increased relative abundance of the genus *Bifidobacterium* (29.6%) when compared to baseline (17.8%) or the samples with MCC (23.1%).

RS had the largest impact on the relative amount of Bifidobacteriaceae (48% vs. 23.3% with MCC). Bifidobacteria together with Lachnospiraceae and Ruminococcaceae, the families harboring major butyrate producers, constituted almost 90% of the total bacterial population. The increase in the relative amount of Lachnospiraceae was mostly due to the genus *Roseburia*, which after 48 h fermentation accounted for 16.1% of the total bacteria (Figure S1). 

### 2.3. Fecal Fermentations of Experimental Dietary Fiber Preparations

**Figure 2:**
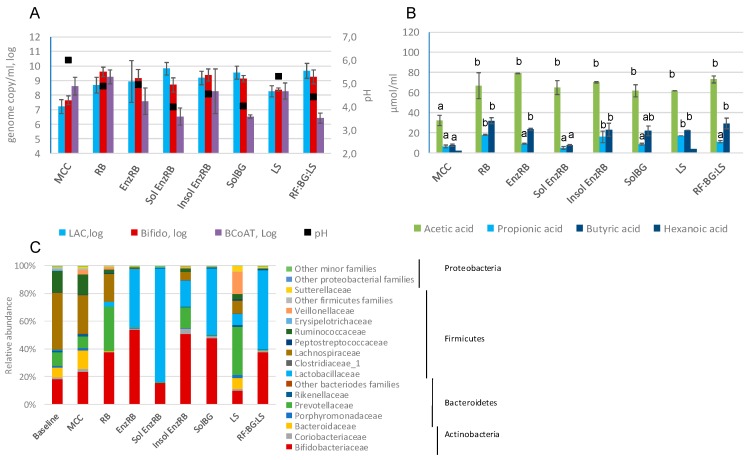
The bacterial population changes and SCFA accumulation after 48 h fermentation of in-house dietary fiber preparations. (**A**) The number of main microbial groups as defined by qPCR; (**B**) the concentration of SCFAs at the end on the fermentation; (**C**) the bacterial community profile at the family level. LAC—*Lactobacillus* group, Bifido—*Bifidobacterium* spp., BCoAT—butyryl-CoA:acetate CoA-transferase gene; MCC—microcrystalline cellulose, LS—linseed, RB—rye bran, EnzRB—enzymatically treated rye bran, Sol EnzRB—soluble fraction of enzymatically treated rye bran, Insol EnzRB—insoluble fraction of enzymatically treated rye bran, SolBG—soluble oat fiber preparation, RF:BG:LS—a mixture of rye fiber:soluble oat fiber preparation:linseed as the ratio of 40:40:20. Values with different letters showed significant differences among the groups (*p* < 0.05).

Seven experimental dietary fiber samples (Table 1) were tested in the in vitro model. All fiber preparations increased the amount of *Lactobacillus* group compared to MCC after 48 h fermentation (Figure 2A). The highest increase of *Lactobacillus* group was seen with soluble oat fiber preparation (SolBG), soluble rye bran fraction (Sol EnzRB) and a mixture of rye fiber, soluble oat fiber preparation and linseed sample (RF:BG:LS). At the same time, these fibers led to the largest drop in the level of butyrate-producing bacteria harboring butyryl-CoA:acetate CoA-transferase gene. No significant differences were seen in the total amount of bifidobacteria. 

All fibers led to increased production of total SCFA, even though to different extents (Figure 2B). All fibers resulted in a significant increase in acetic acid (*p* < 0.05) compared to MCC. Three fibers, rye bran (RB), its insoluble fraction (Insol EnzRB) and linseed samples (LS), induced the accumulation of propionic acids, whereas rye bran preparation (RB), EnzRB and Insol EnzRB, as well as the mixture RF:BG:LS led to an increased level of butyric acid (*p* < 0.05). 

Community composition analyses revealed that EnzRB, Insol EnzRB, SolBG and RF:BG:LS had a clear bifidogenic effect (37.4–53.9% vs. 23.3% with MCC) (Figure 2C and Figure S1A,B). All the tested experimental fiber preparations promoted the growth of lactobacilli, although RB led to a rather small increase (3.4% of the total bacteria). By the end of the fermentation with Sol EnzRB, SolBG and RF:BG:LS, the relative amounts of the genus *Lactobacillus* reached >80%, 47%, and 57%, respectively, of the total bacterial population. 

Prevotellaceae was enriched by RB, Insol EnzRB and linseed. The abundance of Lachnospiraceae decreased with all fibers but still remained at a high level (20.5%) with RB. This decrease was mostly due to a decrease in *Blautia* levels (<1% in the samples with the fibers vs. >5% in the control). At the same time the proportion of *Roseburia* was higher after the fermentation of RB and Insol EnzRB (13.1% and 3.1% respectively) compared to the controls without fibers or with MCC (<1%).

The LS sample led to a notable increase in the relative level of Veillonellaceae, which was entirely represented by the genus *Dialister* (15.9% vs. 2.9% in MCC). The LS sample also increased the abundance of Sutterellaceae (4% of the total bacteria), while in the samples supplemented with all other fibers the level of proteobacteria was at the level of less than 1.5%.

### 2.4. Fecal Fermentations of Dietary Fiber Preparations in the Presence of Amoxicillin-Clavulanate

Changes in the microbial groups in the in vitro model were studied at the presence of amoxicillin-clavulanate and the eleven dietary fiber preparations: Five commercial (MCC as a control fiber) and seven experimental fibers. Amoxicillin-clavulanate treatment resulted in a slight decrease in the total level of the *Lactobacillus* group with RB, all RB-derived fibers and RF:BG:LS mix (Table S1). At the same time, the level of bifidobacteria estimated by qPCR increased with several fibers or remained at the same level as the fermentations without amoxicillin-clavulanate. 

**Figure 3:**
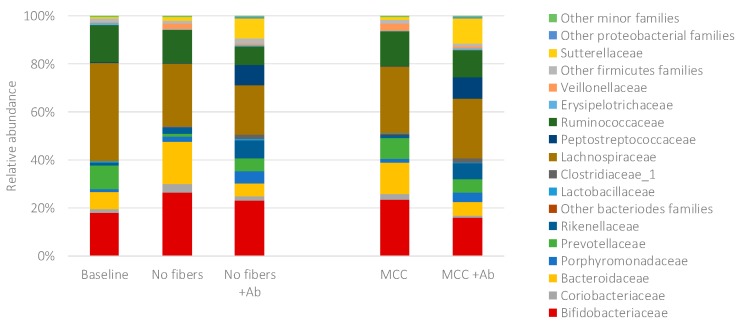
The bacterial community profile at family level in the control samples (baseline, no fibers, microcrystalline cellulose) without and with amoxicillin-clavulanate (Ab) after 48 h of incubation. MCC—microcrystalline cellulose; Ab—amoxicillin-clavulanate.

Amoxicillin-clavulanate affected the overall bacterial community structure. The changes within the bacterial community in the control samples due to exposure to amoxicillin-clavulanate are presented in Figure 3. In the control with MCC, the antibiotic led to a decrease in the relative abundance of Bifidobacteriaceae (23.3% with no antibiotic vs. 15.8% with Ab), Bacteroidaceae (13.35% vs. 5.5%), Prevotellaceae (8.5% vs. 5.6%), Lachnospiraceae (27.6% vs. 24.9%) and Ruminococcaceae (14.2% vs. 11.3%). 

In the samples supplemented with the fibers and amoxicillin-clavulanate, the changes within the population were fiber dependent (Figure S2). The relative abundance of the genus *Bifidobacterium* was not affected by the antibiotic in the samples with XOS and inulin, whereas its abundance decreased in the samples with Sol EnzRB and linseed, and increased in the samples with RS, SolBG, RB, EnzRB and RF:BG:LS mixture (Figure S2B). Genus *Lactobacillus* was not affected by amoxicillin-clavulanate during the fermentation of XOS, SolBG or linseed. The rest of the fibers, however, resulted in a notable drop in the relative amounts of *Lactobacillus* compared to the fermentations without antibiotic (Figure S2D). Genus *Roseburia* was enriched by RB and Insol EnzRB without antibiotic but was almost eliminated with the addition of amoxicillin-clavulanate, even in the presence of the fibers. 

**Figure 4:**
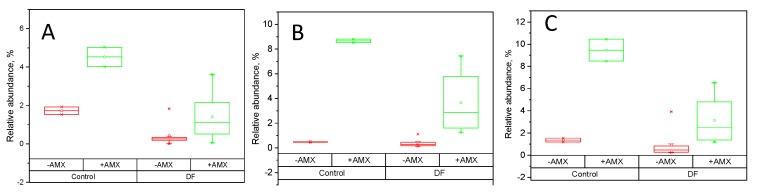
The relative abundance of selected families in the samples with (+AMX, green) and without (−AMX, red) amoxicillin-clavulanate after 48 h of fermentation of RS and experimental fiber preparations. (**A**) Porphyromonadaceae (p_Bacteroidetes), (**B**) Peptostreptococcaceae (p_Firmicutes), (**C**) Sutterellaceae (p_Proteobacteria).

Amoxicillin-clavulanate resulted in the significant increase of Porphyromonadaceae, Peptostreptococcaceae and Sutterellaceae (Figure 4). At the presence of RS and in-house prepared fibers amoxicillin-clavulanate still enriched these bacterial groups, but the increase was smaller than in the control. 

## 3. Discussion

The approach in the study was first to validate the batch-type in vitro colon model for screening purposes using commercial dietary fiber preparations. The in vitro model presented in this study is developed from the batch distal colon model [31] and based on the work of Barry et al. [32]. To minimize the effect of individual variation in the fecal microbiota, a slurry prepared from pooled fecal samples from five healthy adult donors was used. The suitability of pooled fecal samples in fermentation models had been shown earlier [33,34]. Secondly, the model was tested to compare the effects of several fiber samples on the microbiota and production of SCFA in the presence of amoxicillin-clavulanate. 

Resistant starch (RS) used to validate the model enriched three genera *Bifidobacterium*, *Ruminococcus* and *Roseburia*. Multiple in vivo and in vitro studies have shown that bifidobacteria ferment well resistant starches resulting in increased production of butyric acid due to cross-feeding between bifidobacteria and butyrate-producing bacteria [35,36,37,38].

In our model, the family Lactobacillaceae dominated the bacterial community in inulin fermentation. Inulin enriched bifidobacteria, but the relative abundance of Lactobacillaceae was higher than that of bifidobacteria. In the human gut, the inulin-type fructans have been shown to shape the microbial community by increasing *Bifidobacterium* spp., *Faecalibacterium prausnitzii* and decreasing Lachnospiraceae [39,40,41,42]. Recent in vitro study utilizing static fermentation model has shown that inulin enriched bifidobacteria, while *Lactobacillus* group was also increased but to a much lower degree [43]. This model, however, was run for 24 h, while that in the current study for 48 h. Since lactic acid bacteria tolerate acidic conditions well the effect of pH on the bacterial composition cannot be excluded in current work. The structure of inulin-type fructans, particularly the level of polymerization, could also influence the ability of bifidobacteria to utilize them a carbon source [44,45,46]. In vitro fecal fermentation of inulin (the same commercial preparations in the present study) showed an increase of lactic acid bacteria and a decrease in pH in 48 h of fermentation of inulin [47].

Rye bran is reach in arabinoxylans and considered to be a valuable source of dietary fibers [8,48]. Compared to wheat bran, rye bran has more soluble fibers, including arabinoxylans [8]. Linseed is also characterized by a high level of dietary fibers (about 30%), of which one third are water-soluble, including arabinoxylans [49]. 

All dietary fiber samples, prepared in the current study, except for the soluble fraction of enzymatically treated rye (Sol EnzRB) and linseed, had the bifidogenic effect. The bifidogenic capacity of AXOS has been shown in multiple studies and is well outlined in several reviews [50,51,52]. However, bifidobacteria, as shown with pure cultures, have specific preferences to different types of AXOS [44,53,54]. Enzymatic hydrolysis of the rye bran was expected to increase the proportion of soluble fiber, especially AXOS, since xylanase was one of the main enzymatic activities in the used enzyme mixture. It turned out that insoluble fiber content was actually clearly increased. These insoluble fibers could affect the bacterial community and overall fermentation. It has been shown for wheat bran preparations, that only a particular part of the luminal bacterial population colonized the bran particles [55,56]. The enzyme mixture contained also amylase, which resulted in the formation of oligosaccharides other than AXOS. The high amount of free sugars and non-arabinoxylan oligos in some the rye bran preparations could potentially mask the impact of AXOS present. Thus, in the future studies, pure enzyme preparations and removal of free sugars should be performed first when the specific effects of the modified dietary fiber preparations are studied. Further, more detailed characterization of the molecular weight of the soluble fibers would be beneficial to reveal the effects of the degree of polymerization of specific fibers on the microbiota. 

Regarding linseed preparation, the only treatment applied was defatting, which did not remove proteins and lignans. Therefore, the availability of dietary fibers from linseed to gut microbiota could be improved by physicochemical and enzymatic treatments [57] that were used for rye bran.

Generally, when applying fiber preparations with complex compositions and structures, drawing very specific conclusions is always challenging, but these preparations are still relevant when considering feasible industrial-scale production of dietary fiber preparations and human consumption. 

The mixture of three different fibers, rye fiber, soluble oat fiber, and linseed sample (RF:BG:LS), was tested since it has high fiber variation and availability of both highly soluble and insoluble fibers. This mixture had also the bifidogenic effect due to the high fraction of rye bran and beta-glucan. The fermentation of the mixture RF:BG:LS resulted in butyrate accumulation, while the level of butyrate-producing bacteria, estimated by qPCR of butyryl-CoA:acetate CoA-transferase (BCoAT) gene, was lower compared to the control. It is generally accepted that the main pathway of the butyrate production in human colon involves mostly BCoAT activity and to a smaller extent butyrate kinase [58]. Moreover, a metagenomic analysis of a large number of bacterial genomes, including those derived from human fecal samples, showed that even though the acetyl-coenzyme A (CoA) pathway was the dominant accounting for about 80% of all pathways, lysine pathway was found in 11 genomes [38]. Thus, in the present study, we could miss part of the butyrate producers by using only qPCR specific to one particular enzyme.

The second element in the present study was to test the hypothesis that applications of specific dietary fiber preparation will alleviate the effects of antibiotics on the microbiota. Consumption of amoxicillin, commonly used β-lactam antibiotic, often results in a high rate of antibiotic-associated diarrhea [59,60]. The negative effect of amoxicillin on fecal bacterial communities has been documented [61,62,63]. In the current study, amoxicillin was used together with clavulanate which inhibits bacterial β-lactamase activity. These two drugs are often administered together. The samples with no fibers or that with MCC amoxicillin-clavulanate resulted in a shift within the bacterial population by reducing the relative abundance of bifidobacteria and increasing that of proteobacteria (family Sutterellaceae). The family Sutterellaceae has been associated with intestinal inflammation [64].

In vivo studies of patients of different ages showed that amoxicillin treatment affected the diversity of bifidobacterial species [61,62]. The species affected were *B. adolescentis, B. bifidum and B. catenulatum* group but not *B. longum*. Since we focused on taxonomic resolution at genus and family level (due to the low resolution of the V3-V4 region for several bifidobacterial species), no data on the effect of amoxicillin on particular bifidobacterial species in our model are available. Metagenomic analyses of fecal samples from adults on amoxicillin therapy revealed that the main groups negatively affected by amoxicillin treatment were the families Coriobacteriaceae, Peptostreptococcaceae, Lachnospiraceae, and Ruminococcaceae [63]. In the present study, however, it was found that Peptostreptococcaceae was increased by amoxicillin in the control without fibers.

The next step in the present study was to investigate whether dietary fiber preparations could potentially counteract the effects of amoxicillin on the microbiota. The effect of amoxicillin along with doxycycline and clindamycin on the gut microbiota as well as the potency of FOS and two types of XOS to minimize their negative effect has been investigated using in vitro static batch fermentation model similar to that we established [15]. The authors also found that amoxicillin affected the microbiota in fiber dependent manner. In the current study, we also found that different dietary fiber preparations had different effects on the bacterial populations in the presence of the antibiotic. Genus *Roseburia*, for instance, was enriched by RB preparation as well as its soluble fraction but was eliminated by antibiotic even in the presence of these fibers. In the presence of enzymatically treated RB and its insoluble fraction, amoxicillin-clavulanate targeted mostly genus *Lactobacillus*. In the samples with dietary fiber preparations, the increase in the relative levels of proteobacterial family Sutterellaceae due to antibiotic treatment was much smaller compared to the controls without fibers. Thus, dietary fiber preparations tested in the current study have the potential to mitigate antibiotic-associated disturbances in the human microbiota. Additional study, however, is needed to understand which particular fractions of the fiber preparations have a protective effect of the commensal gut microbes. 

## 4. Materials and Methods 

### 4.1. Fibers and Their Characterization

Twelve different dietary fiber containing samples were used in the study. The composition of the fiber samples that were used as such are listed in Table 2. 

In addition, seven fiber samples were prepared by different methods to obtain a range of samples with varying composition. Linseed ingredient (FlaxseedFields^®^ Natural, Linseed Protein Finland Ltd., Kauhajoki, Finland) was defatted in a Soxlet equipment by using hexane in order to increase the content of dietary fiber in the sample, followed by grinding. Rye bran (Fazer Mills, Lahti, Finland) was dry fractioned in order to reduce the content of starch and increase the content of dietary fiber in the sample. The bran was first dry milled twice using a 100UPZ pin disc mill (Hosokawa-Alpine, Augsburg,, Germany) at a rotor speed of 17800 rpm, followed by air classification in four batches of approximately 1.1 kg with Minisplit air classifier (British Rema Manufacturing Company Ltd., Chesterfield, UK) using air classifier wheel speed of 3000 rpm and air flow of 220 m^3^/h. The coarse fraction (average mass yield 38 ± 2%) from the air classification (RB) was further treated with a mixture of commercial hydrolytic enzyme preparations (Depol 740 L, Biocatalysts Ltd., Wales, UK, dosage 11 µL/g bran, corresponding to 200 nkat xylanase activity/g bran; Veron CP, AB Enzymes GmbH, Darmstadt, Germany, dosage 13 mg/g bran, corresponding to 100 nkat xylanase activity/g bran; and Grindamyl A14000, Danisco, Copenhagen, Denmark, dosage 0.6 mg/g bran, corresponding to 75 nkat α-amylase activity/g bran) for 4 h at 40 °C at 18% bran concentration. After the treatment, the mixture was directly freeze-dried and ground to produce enzymatically treated rye bran (EnzRB) or centrifuged to separate the supernatant and residue, which were freeze-dried to produce the soluble (Sol EnzRB) and insoluble (Insol EnzRB) fractions of the enzymatically treated rye bran, respectively. Soluble oat beta-glucan (SolBG) was produced from oat bran concentrate (Aurora oat beta-glucan 30, Fazer Mills, Finland) according to the procedure described elsewhere [65] with modifications. Briefly, oat bran concentrate was treated with a hydrolytic enzyme preparation (Depol 740L, Biocatalysts Ltd., dosage 5 nkat beta-glucanase activity/g bran) in an extruder at low water content (approx. 60%) and incubated for 2 h at 50 °C, after which the enzyme was inactivated and the sample extracted by adding boiling water followed by homogenization. The homogenized material was centrifuged, and the supernatant was freeze-dried. A sample consisting of a mixture of SolBG, Faze Rye fiber (Fazer Mills, Finland) and defatted (by supercritical CO2 extraction) linseed was prepared by mixing the ingredients in a weight ratio of 40:40:20, respectively.

### 4.2. The In Vitro Fermentation Static Model

#### 4.2.1. Preparation of Fecal Inoculum

Fecal samples were obtained from five healthy adult volunteers. The samples were collected into a tightly closed box with an oxygen-consuming pillow (Anaerocult Mini; Merck, Darmstadt, Germany) and used within two hours of collection. Inoculum preparation and further fermentations were conducted under strictly anaerobic conditions in an anaerobic cabinet (10% H_2_; 10% CO_2_; 80% N_2_). For each of the four experiments, the donor delivered fresh fecal samples. 

Fermentation medium was based on ileal efflux medium SIEM [66] simulating of adult chyme entering the colon. For inoculant preparation, equal amounts of fecal material from all donors were pooled and 10% (*w*/*v*) fecal suspension was prepared in SIEM two times diluted with a buffer. SIEM had the following composition (per liter): Pectin, xylan, arabinogalactan and amylopectin 9.4 g of each, casein—47.0 g, starch—78.4g, tween 80–34.0 g, bactopeptone—47.0 g, and bile—0.8 g. The composition of the buffer was (per liter): 2.5 g K_2_HPO_4_×3H_2_O, 1.5 g KH_2_PO4, 2.5 g NaHCO_3_, 4.5 g NaCl, 0.005 g FeSO_4_×7H_2_O, 0.5 g MgSO_4_×7H_2_O, 0.45 g CaCl_2_×2H_2_O, 0.05 g bile, and 0.4 g cysteine hydrochloride, and 1 mL of a vitamin mixture containing (per liter): 1 mg menadione, 2 mg D-biotin, 0.5 mg vitamin B12, 10 mg pantothenate, 5 mg nicotinamide, 5 mg p-aminobenzoic acid, and 4 mg thiamine. The obtained suspension was homogenized in Warring Blender two times for 15 s and filtered through a 1 mm sieve to remove large particles. After 2-h pre-incubation, the 10% fecal suspension was further diluted 1/10 (*v*/*v*) with the buffer. 

#### 4.2.2. Experimental Setup 

The in vitro static fermentation was performed in a 24-well microtiter plate under anaerobic conditions with pH recording at the beginning and at the end on the fermentation. Tested fibers were placed into wells one day prior to the experiments and plates were kept in a desiccator at room temperature. On the day of the experiment, the plates were transferred into the anaerobic cabinet. 

2 mL of the prepared fecal inoculum was added to each well containing 20 mg of dry fiber preparations and mixed carefully. Microtiter plates were kept on a shaker with very low agitation. Fermentations were carried out at 37 °C. After 24-h incubation, the samples were transferred to new plates containing a fresh portion of the fibers (20 mg/well). Each fiber preparation was checked in duplicate and the experiments were performed three-four times using freshly collected fecal samples. The fiber fermentations with or without the antibiotic were carried out simultaneously using the same fecal inoculant. As a fiber control, microcellulose (MCC) was used, since cellulose is generally considered to be poorly degraded by in human gut microbiota [67]. The initial pH was set to 6.8 and the pH in each well was measured at the end of the experiments. 

When the effect of the antibiotic on the fecal microbiota was studied, amoxicillin-clavulanate was added into the wells. A stock solution of amoxicillin at a concentration of 50 mg/mL was prepared in 1N NH_3_OH on the day of the experiment. A stock solution of potassium clavulanate at a concentration of 50 mg/mL was prepared in sterile water. Sixteen µL of the amoxicillin stock and 4.8 µL of potassium clavulanate stock were added into wells to obtain the final amoxicillin concentration of 400 µg/mL. The same amounts of 1N NH_3_OH and sterile water were added into wells without antibiotic. The addition of 1N NH_3_OH also helped to prevent the drop in pH during the fermentation. 

The rationale behind the high amoxicillin concentration was that amoxicillin belongs to the Class III (high solubility, low permeability) pharmaceuticals in the Biopharmaceutical Classification System. The fraction absorbed after oral administration of amoxicillin is estimated to be about 88% [68]. Taking in account the therapeutic dosage of 750–1750 mg/day and the average amount of feces of 130 mL/day [69], the concentration of amoxicillin may reach the level of about 600–1.5 mg/g of feces.

### 4.3. Analyses of Fecal Samples 

#### 4.3.1. Short Chain Fatty Acid Analysis

The samples were thoroughly vortexed before centrifuging at 10,000 rpm for 10 min. From the clear supernatant, 300 µL aliquots were taken for the extraction process. The samples were spiked with 40 µL of an internal standard mixture containing 104.5 µg and 408.3 µg heptanoic acid (C7) and C13 labeled butanoic acid (C4), respectively, and acidified with 6M hydrochloric acid (100 µL). Diethyl ether was used for extraction (3 mL) and after vortexing (15 min) and standing (15 min) part of the organic phase was transferred into a GC vial. 

Quantification was based on calibration curves for acetic (C2), propionic (C3), isobutyric (i-C4), butyric (C4), isovaleric (i-C5), valeric (C5) and hexanoic (C6) acids. Calibration curves were prepared with seven concentrations the most dilute varying from 1.7–20.1 µg/sample (0.01–0.33 mM) and the most concentrated from 0.205–2.410 mg/sample (1.77–40.13 mM). 

The samples (1 µL) were run in splitless mode by Agilent GC-MS equipped with an HP-INNOWax silica capillary column (60 m × 250 µm × 0.25 µm). The oven temperature program was from 40 °C (1.5 min) to 200 °C at a rate of 15 °C/min and then to 240 °C at a rate of 50 °C. The total run time was 23 min. The MS source and quadrupole temperatures were 230 and 150 °C, respectively, and the data were collected from *m*/*z* 30 to 600.

#### 4.3.2. DNA Extraction

DNA was extracted from 0.2 mL sub-samples. Before the DNA extraction, the samples were treated with Propidium monoazide (PMA™ dye, Biotium Inc., Hayward, CA, USA). The PMA stock solution (2 mM) was prepared in water. Ten microliters of the stock were added to 0.2 mL of the fecal slurry to obtain a final concentration of 100 μM. This step was performed in the anaerobic cabinet. The treatment was performed according to the manufacturer’s instruction: Tubes were incubated for 10-min in the dark on a shaker and PMA was photoactivated for 15 min in a PMA-Lite™ LED Photolysis Device (Biotium). After PMA treatment, the samples were centrifuged at 12,000× *g* for 10 min and the supernatant was replaced with pure water. The samples were stored at −80 °C until DNA extraction. 

Genomic DNA was extracted using the FastDNA SPIN Kit for Soil (MP Biomedicals, USA) according to manufacturer’s instructions with small modifications. The amount of extracted DNA was quantified using the NanoDrop 2000c (Thermo Scientific, Wilmington, DE, USA). The DNA was stored at −80 °C until used.

#### 4.3.3. Bacterial Quantification Using Real-Time qPCR (qPCR)

The bacterial number was estimated by qPCR with the primers specific for total bacteria, *Lactobacillus*-groups and *Bifidobacterium* as described elsewhere [70]. The abundance of butyrate producers was estimated by qPCR specific for butyryl-CoA:acetate CoA-transferase gene using primers developed by Louis and Flint [71]. The reaction (20 µL) consisted of 2× Master Mix (High Resolution Melting Master Kit, Roche, Mannheim, Germany), 2 mM MgCl_2_, 2.5 µM primers, and 5 µL of template DNA (5 ng/reaction). The thermal cycling condition was as follows: 10 min at 95 °C hot-start, 45 cycles of denaturation at 95 °C for 10 s, annealing at 53 °C for 20 s and extension at 72 °C for 35 s, followed by melting curve analyses. A standard curve was obtained from genomic DNA of *Roseburia intestinalis* VTT E-052785^T^. 

All qPCR reactions were performed on the LightCycler 480 System (Roche Diagnostics, Basel, Switzerland), and the results were analyzed with the LightCycler 480 software version 1.5 (Roche).

For each bacterial group of interest, the number of genomes in the standards was calculated based on the genome size identified through the Center for Biotechnology Information genome database.

#### 4.3.4. 16S rRNA Gene Amplicon Sequencing

Bacterial community structure was assessed by sequencing of the V3–V4 region within the 16S rRNA gene. Extracted total DNA was sent to Microsynth AG for library preparation and sequencing. Sequencing was done on the Illumina MiSeq sequencing platform with the V3 Illumina kit (2 × 300 bp paired-end reads). The V3–V4 region of the bacterial 16S rRNA gene was amplified with the set of primer pair 341F/785R. 

Sequencing data were processed with Mothur package version 1.4.1 [72] according to SOP. Briefly, paired-end sequences were merged into contigs to give the assembled sequences of a minimum length of 400 bp, followed by removal of the reads with ambiguous bases and homopolymers longer than 8 bp. The sequences were aligned against Mothur-adapted SILVA bacterial reference database [73] and screened for chimeras using Mothur incorporated UCHIME in *de novo* mode. Quality-filtered sequences were classified to taxa using the implemented Mothur RDP Naïve Bayesian rRNA Classifier (trainset 16) [74] with an 80% confidence. The sequences not classified as bacterial—plant chloroplasts, mitochondria, and unknown sequences—were omitted from the downstream analyses. The sequences were grouped to OTUs at the 97% similarity. 

The reads were sub-sampled to 57,210 reads/sample and the results of relative abundance are presented based on the sub-sampled data. 

#### 4.3.5. Statistical Analysis

Results of qPCR and SCFA accumulation are reported as mean ± SEM. SCFA accumulation data were tested for normality using the Shapiro-Wilk test. If normally distributed, differences between groups were evaluated using one-way ANOVA (Tukey’s tests, *p* < 0.05). The analyses were performed using Origin software (version 2019, OriginLab Corporation, Northampton, MA, USA).

## 5. Conclusions

In the present study, we showed that the simple microtiter format in the in vivo fermentation model can be used for the screening of the dietary fiber preparations on gut microbiota. While applying the fermentation model, special care should be taken to avoid the presence of easily-fermented carbohydrates in the preparations, which could mask the beneficial effect of the fiber preparations. 

Our study showed that the new experimental fiber preparations based on the rye and linseed had prebiotic potential. Several new fiber preparations were able to mitigate antibiotic-associated disturbances in the human gut microbiota by slowing the increase of proteobacteria and supporting the growth of bifidobacteria. More detailed characterization of these fiber preparations and additional validations in the in vivo model are needed to reveal the specific mechanism behind why these fibers caused the observed effect.

## Figures and Tables

**Table 1:** The compositions of processed fiber samples.

Sample ^1^	Dietary Fiber ^1^, % (as is)				Protein ^2^, %	Digestible Starch and Available Carbohydrates (% as Is) ^2^
	Insoluble ^2^	Soluble ^2^	Oligos ^3^	Total ^4^		
LS	25.7 ± 0.1	14.9 ± 0.1	1.4	42.0	29.7 ± 0.1	0.1 ± 0.1
RB	35.5 ± 0.1	6.7 ± 0.0	7.0	49.2	15.2 ± 0.2	14.6 ± 0.3
EnzRB	25.3 ± 0.3	4.9 ± 0.2	11.4	41.6	14.8 ± 0.3	16.8 ± 0.1
Sol EnzRB	0.0 ± 0.0	6.6 ± 0.8	25.5	32.1	11.1 ± 0.1	28.1 ± 0.2
Insol EnzRB	37.1 ± 0.3	6.2 ± 0.0	8.7	51.9	16.3 ± 0.0	12.9 ± 0.2
SolBG	1.1 ± 1.1	52.3 ± 0.8	6.4	59.8	11.6 ± 0.3	11.9 ± 0.0
RF:BG:LS	16.9 ± 0.6	25.3 ± 0.3	4.9	47.1	15.5 ± 0.1	12.0 ± 0.0

^1^ LS—linseed, RB—rye bran, EnzRB—enzymatically treated rye bran, Sol EnzRB—soluble fraction of enzymatically treated rye bran, Insol EnzRB—insoluble fraction of enzymatically treated rye bran, solBG—soluble oat fiber preparation, RF:BG:LS—a mixture of rye fiber:soluble oat fiber preparation:linseed as the ratio of 40:40:20. ^2^ Results are presented as the mean of two duplicates ± average deviation. ^3^ Oligos were analyzed without duplicates. ^4^ Total dietary fiber is calculated as the sum of insoluble, soluble and oligos.

**Table 2:** Commercial/Isolated fiber samples (data from the supplier).

Fiber	Short Name	Supplier	Product	Specifications
Microcrystalline Cellulose	MCC	JRS Pharma	VIVAPUR^®^ 105	Average particle size by laser diffraction 15 µm
Resistant Starch	RS	Ingredion	HI-MAIZE^®^ 260	Dietary fiber 56%, other carbohydrates 31%
Xylo-oligosaccharide	XOS	Shandong Longlive Bio-Technology	Xylo-oligosaccharide	Purity >95%
Inulin	Inulin	Beneo	Orafti^®^ HSI	Inulin content ~88%

## Supplementary Materials

Supplementary materials can be found at https://www.mdpi.com/1422-0067/20/8/1925/s1.
