# Supplementary material for: A Small In Vitro Fermentation Model for Screening the Gut Microbiota Effects of Different Fiber Preparations
Source: Int J Mol Sci. 2019 Apr 18;20(8):1925. doi: 10.3390/ijms20081925 (PMC6514940; doi:10.3390/ijms20081925)
Supplement: Supplementary file 1 [file ijms-20-01925-s001.pdf]

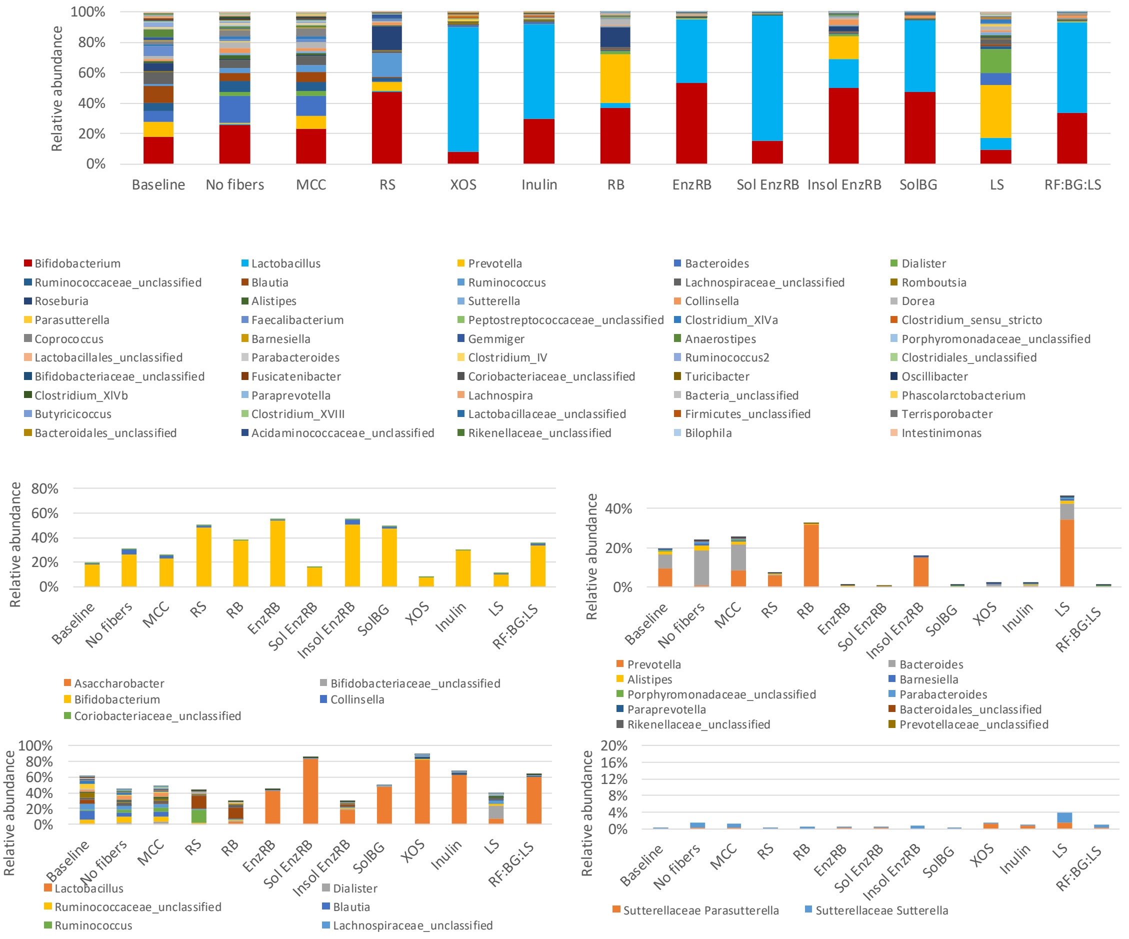

Figure S1. The composition of bacterial community (genus level) after 48-h fermentation: (A) the major 60 genera; (B-E) main genera within phyla Actinobacteria (B), Bacteroidetes (C), Firmicutes (D) and Proteobacteria (E). MCC - microcrystalline cellulose, RS - Resistant Starch, LS - linseed, RB - rye bran, EnzRB - enzymatically treated rye bran, Sol EnzRB - soluble fraction of enzymatically treated rye bran, Insol EnzRB - insoluble fraction of enzymatically treated rye bran, SolBG -soluble oat fiber preparation, RF:BG:LS - a mixture of rye fiber:soluble oat fiber preparation:linseed at the ratio of 40:40:20.

Table S1. The number of selected microbial groups after 48-h fermentation of the dietary fiber samples with and without omaxicillin-clavulanic acid as determined by qPCR. For clarity the samples (fermentation of the fibers without antibiotic) presented on Figure 2 are added to this table.

Dietary fiber preparation	Bacterial number ± StDev			
	LAC	BCoAT	Bifido	pH
Baseline	7.2±0.5	8.6± 0.1	7.6±0.81	6.8±0.1
No fibers	7.5±0.4	8.5±0.4	9.0±0.3	6.1±0.1
No fibers + Ab	7.5±0.8	8.44±0.4	9.3±0.61	6.2±0.13
MCC	7.5±0.5	8.4±0.6	8.8±0.3	6.0±0.2
MCC +Ab	7.4±0.9	8.09±0.7	9.2±0.86	6.0±0.1
RS	7.7±0.4	9.9±0.6	9.8±0.2	4.7±0.2
RS +Ab	7.4±0.2	9.65±0.4	9.5±0.22	4.7±0.1
XOS	8.5±0.6	6.0±0.6	8.5±0.4	3.9±0.1
XOS+Ab	8.5±0.6	6.31±1.2	9.09±0.40	3.8±0.2
inulin	9.5	6.9	9.2	4
inulin+Ab	9.5	6.37	9.36	4.1
RB	8.7±0.5	9.3±0.5	9.6±0.3	4.9±0.1
RB+Ab	7.9±1.2	7.6±1.1	9.0±0.40	4.9±0.2
EnzRB	8.9±1.4	7.6±0.9	9.2±0.6	4.9±0.7
EnzRB +Ab	7.6±1.5	7.8±1.1	9.8±0.69	5.0±0.7
Sol EnzRB	9.8±0.4	6.5±0.6	8.7±0.5	4.0±0.2
Sol EnzRB +Ab	8.5±0.8	6.2±0.36	9.1±0.6	4.0±0.1
Insol EnzRB	9.2±0.5	8.3±1.5	9.3±0.4	4.5±0.2
Insol EnzRB+Ab	8.0±.19	8.3±0.88	9.7± 0.5	4.8±0.2
SolBG	9.5±0.4	6.5±0.1	9.1±0.2	4.0±0.1
SolBG+Ab	8.7±0.6	6.4±0.26	9.6±0.6	4.3±0.1
LS	8.3±0.4	8.3±0.5	8.4±0.1	5.2±0.1
LS+Ab	8.3±.19	8.28±1.70	9.4±0.7	5.3±0.1
RF:BG:LS	9.7±0.5	6.4±0.3	9.3±0.5	4.4±0.2
RF:BG:LS +Ab	8.5±1.0	7.18±	9.2±0.27	4.4±0.1

LAC - *Lactobacillus*-group; Bifido - *Bifidobacterium* spp.; BCoAT - butyryl-CoA:acetate CoA-transferase gene; MCC - microcrystalline cellulose, RS - resistant starch, LS - linseed, RB - rye bran, EnzRB - enzymatically treated rye bran, Sol EnzRB - soluble fraction of enzymatically treated rye bran, Insol EnzRB - insoluble fraction of enzymatically treated rye bran, SolBG -soluble oat fiber preparation, RF:BG:LS - a mixture of rye fiber:soluble oat fiber preparation:linseed at the ratio of 40:40:20.

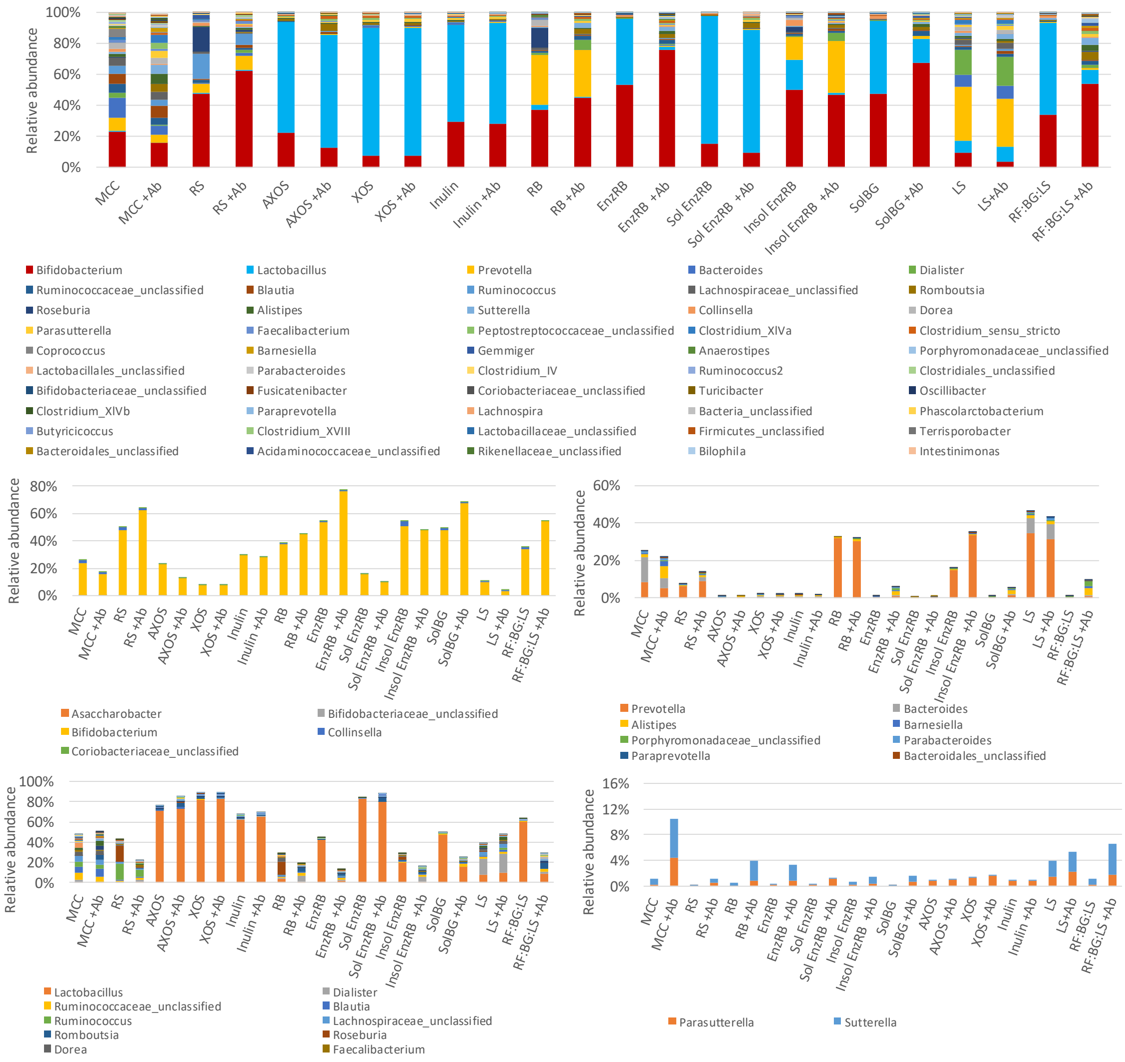

Figure S2. Changes in the bacterial community during the fermentation of the fibers without and with amoxicillin-clavulanate: (A) the major 60 genera; (B-E) main genera within phyla Actinobacteria (B), Bacteroidetes (C), Firmicutes (D) and Proteobacteria (E). For clarity the samples (fermentation of the fibers without ampoxicillin-clvulanate) presented in Figure S2 are added to this figure. MCC - microcrystalline cellulose, RS - resistant starch, LS - linseed, RB - rye bran, EnzRB - enzymatically treated rye bran, Sol EnzRB - soluble fraction of enzymatically treated rye bran, Insol EnzRB - insoluble fraction of enzymatically treated rye bran, SolBG -soluble oat fiber preparation, RF:BG:LS - a mixture of rye fiber:soluble oat fiber preparation:linseed at the ratio of 40:40:20.
